# Managing long COVID symptoms and accessing health services in Brazil: A grounded theory analysis

**DOI:** 10.1016/j.heliyon.2024.e28369

**Published:** 2024-03-22

**Authors:** Francielle Renata Danielli Martins Marques, Carlos Laranjeira, Lígia Carreira, Adriana Martins Gallo, Wanessa Cristina Baccon, Herbert de Freitas Goes, Maria Aparecida Salci

**Affiliations:** a Departamento de Pós-Graduação em Enfermagem, Universidade Estadual de Maringá, Avenida Colombo, 5790—Campus Universitário, Maringá, 87020-900, PR, Brazil; b School of Health Sciences, Polytechnic University of Leiria, Campus 2, Morro do Lena, Alto do Vieiro, Apartado 4137, 2411-901, Leiria, Portugal; c Centre for Innovative Care and Health Technology (ciTechCare), Polytechnic University of Leiria, Campus 5, Rua das Olhalvas, 2414-016 Leiria, Portugal; d Comprehensive Health Research Centre (CHRC), University of Évora, 7000-801, Évora, Portugal

**Keywords:** COVID-19, Long COVID, Healthcare access, Experiences, Grounded theory, Brazil

## Abstract

**Background:**

The worldwide community has shown significant interest in researching the management of Long COVID. However, there is scarce evidence about the daily experiences of people living with Long COVID and their insights into the healthcare services provided to them.

**Aims:**

This study aims to understand the experience of Long COVID sufferers with their symptoms and in accessing health services.

**Method:**

We employed Charmaz's grounded theory methodology, informed by constructivism, and applied the COREQ guidelines for qualitative research. Sixty-six Brazilians living with Long COVID participated in the study. The data was collected using semi-structured telephone interviews and analyzed using a constant comparative process.

**Findings:**

The findings refer firstly to the consequences of persistent Long COVID symptoms. Secondly, they describe how the disease trajectory required Long COVID sufferers to reorganize their routines and develop adaptive strategies. Lastly, they reflect a diverse array of both positive and negative interactions inside the healthcare system conveyed by individuals suffering from Long COVID. These elements converge towards the core category of the study: “The limbo of Long COVID sufferers: between the persistence of symptoms and access to health services”.

**Conclusions:**

Long COVID is characterized by its varied nature, including a range of physical and emotional repercussions experienced by individuals. There is a need for enhanced comprehension and discourse about Long COVID across several domains, including the general public, policy-making entities, and healthcare professionals. In this sense, the development of specialized services or the reinforcement of existing services to support long COVID sufferers is imperative.

## 1 Introduction

By the end of June 2023, COVID-19 had a global incidence of 768 million confirmed cases and seven million deaths [1]. In the acute phase, the disease can be asymptomatic, mild, moderate, and severe [2]. However, the multifaceted aspects surrounding COVID-19 after the acute phase reflect traumatic experiences that represent a global challenge [3,4].

The scientific community is committed to producing information on how to deal with the long-term consequences of the disease [5]. Due to the persistence of symptoms, post-COVID-19 syndrome can imply two concepts: Long COVID, described as the continuation or development of new symptoms 12–24 weeks after the disease's acute phase; and persistent COVID, which lasts more than 24 weeks [6]. We will use the Long COVID terminology for all symptoms over 12 weeks, in line with the convention adopted by the World Health Organization (WHO) [7].

Persistent symptoms of COVID-19 appear heterogeneously, meaning patients need regular surveillance. Evidence points to symptoms of fatigue, shortness of breath, post-exertional malaise, coughing, chest pain, headache, sleep problems, reasoning difficulties, dizziness, musculoskeletal pain, depression, and anxiety as the most prevalent in people with Long COVID [[8], [9], [10]]. These symptoms are present in combination and can affect the quality of life [11].

Two meta-analyses present similar results, estimating that the global prevalence of Long COVID reached 54% of survivors who were hospitalized, and highlighting the need to monitor people in the post-discharge period [12,13]. In a study analyzing 439 individuals in Brazil, 84% reported at least one prolonged symptom of COVID-19: fatigue was the most frequent, followed by arthralgia, depression and anxiety, dyspnea, and myalgia [8].

Symptom persistence was associated with patients having worse long-term health status, worse quality of life, and severe psychological distress [5,8]. This demonstrates that the long-term effects of COVID-19 can cause disability, decreased quality of life, hyperutilization of health services, and potentially reduced life expectancy [14,15]. Consequently, current clinical practice has adopted an approach based on managing the symptoms of Long COVID [16].

The consequences of Long COVID go beyond physical symptoms. Available scientific evidence demonstrates a high burden of biopsychosocial challenges [17]. These dimensions involve interventions at different levels to strengthen social and health policies, and reduce socioeconomic disparities, in addition to strengthening people's involvement in decision-making through evidence-based clinical practice [18]. Despite the improved clarity in defining Long COVID criteria, there are knowledge gaps about the help individuals seek or require outside official healthcare systems [19].

Users whose needs go beyond the reach of healthcare systems are often not correctly identified and monitored by health professionals and are considered “hyperusers”, regardless of the number of consultations [20]. The WHO suggests that care for people with persistent COVID-19 symptoms be provided through a comprehensive, person-centered multidisciplinary approach involving basic and specialized services [7]. These services should offer rehabilitation, social support, mental health support, and symptom management so the individual can be treated holistically [21].

The socio-ecological model (SEM) proposed by Bronfenbrenner allows us to understand how Long COVID has affected people's lives beyond physical symptoms. Systemic approaches are recommended to guide public health practices [[22], [23], [24], [25]]. This approach elucidates the impact of an individual's physical, social, and cultural aspects (as well as their political surroundings and personal traits) on their health, well-being, and social cohesiveness [21].

Individuals who exhibit heightened health vulnerability from a biopsychosocial perspective are more susceptible to inadequate coping mechanisms in response to Long COVID's repercussions. As a result, these individuals need more attention and support from healthcare professionals [24]. Previous studies recommended expanding knowledge about the physical and emotional consequences of the disease so that results can improve services to meet the health needs of those involved [17,26,27]. To ensure the provision of optimal treatment within health services, a comprehensive understanding of the lived experiences of those affected by Long COVID is essential, as well as providing them with access to healthcare services [26].

In Brazil's universal healthcare system, most individuals can access healthcare services without charge at the time of service [28]. Nevertheless, the ability to acquire services is hindered by a healthcare system subjected to a prolonged period of financial constraints, budget limitations, escalating waiting periods, strained services, accumulated workloads, and insufficient staffing [29]. The pandemic has compounded these issues, with negative effects on individual access to healthcare services. The COVID-19 pandemic has been described as a complex situation in which several factors (such as social, economic, and health disparities) interact and contribute to exacerbate its effects. When patients undergo transitions across several care pathways, they face fragmented healthcare services. Patients frequently seek consultations with general practitioners who serve as intermediaries for accessing specialized treatments. The intricate nature and lack of certainty about the diagnosis, management, and consequences of Long COVID can potentially impose a strain on the healthcare system [30].

Few international qualitative studies have analyzed the effects of Long COVID and the use of health services [17,26,27,31,32]. Previous research briefly discussed how healthcare professionals (HCPs) did not believe patients with Long COVID (resulting in patients having to manage their symptoms independently [33]) and emphasized the significance of relationship-based care. However, there is a limited critical examination of the experiences of those living with Long COVID who are unable to access sufficient healthcare. Brazil follows this trend, with no known qualitative assessment of people's experience with Long COVID and access to health services. The dearth of effective medical treatments and data to guide practitioners in managing Long COVID highlights the intricate nature of this condition, posing an unparalleled challenge for both patients and healthcare professionals. Patients with Long COVID exhibit apprehension with the lack of information and comprehension about the condition. They also report encountering contradictory or incongruous advice from healthcare professionals [26]. There is a need for research that prioritizes the patient's voice and their health by establishing collaborations that bring patients' interests to the forefront [34].

This study aims to understand the experience of Long COVID sufferers and their interactions in accessing health services. In this way, we sought to answer the following question: How do people with Long COVID perceive their current life situation and how do they access health services? This research hopes to contribute to understanding the repercussions of Long COVID on its sufferers and to produce information about their health needs, allowing for better organization of services based on the new demands generated post-COVID-19.

## 2 Materials and methods

### 2.1 Study design

This study is part of a broader Brazilian project entitled “Longitudinal monitoring of adults and elderly people who were discharged from hospital due to COVID-19” to explore and analyze predictors, sequelae, and repercussions of COVID-19 in adults and elderly people who developed severe illness after hospital discharge [35].

In the current study, we conducted a qualitative inquiry based on Charmaz's Constructivist Grounded Theory (CGT) methodology [36]. CGT is an excellent tool for recognizing “multiple realities, seeking diverse perspectives, and engaging in critical analysis throughout the research process” [37] (p.311). It highlights a researcher's reflexivity, giving them greater analytical power over the data [37].

The present study was carried out and documented following the Consolidated Criteria for Reporting Qualitative Research (COREQ) checklist [38].

### 2.2 Context, participants and recruitment

Brazil was among the countries most affected by the COVID-19 death rate, not only within Latin America but also worldwide, falling just behind the United States [39]. At the same time, Brazil has a profoundly different context in terms of accessibility to health services [8] due to the Brazilian population's high socioeconomic vulnerability [40,41].

The study was carried out in the south of Brazil, in the State of Paraná, which is the sixth most economically important federative unit in the country [42]. Like other states, Paraná had one of the highest transmission rates of COVID-19 requiring hospital admission [43], justifying the recruitment of participants who developed Long COVID in this State.

Participants were selected by purposeful sampling from notification forms available in the Notifica COVID Paraná databases and the Influenza Epidemiological Surveillance Information System (SIVEP-Influenza). Inclusion criteria for eligible participants were: 1) people with one or more long COVID symptoms [Long COVID after 12 weeks of acute infection]; 2) having used health services during the Long COVID phase; 3) having the ability to communicate and understand Portuguese; and 4) having responded to the telephone contact (up to three attempts). Pregnant and postpartum women were excluded.

To obtain maximum sample variation, individuals were recruited regardless of sex, race, or age. As a result, 66 people participated in the study and were organized into two sample groups. The first group was made up of 28 people who only used the public health system. The second group included 38 people who used private health services. This number was determined during conceptual exploration when no new evidence was produced, indicating theoretical saturation [37].

### 2.3 Data collection

Data collection took place from May to August 2022. Telephone contacts were extracted from the registration platforms (Notifica COVID Paraná and SIVEP-Influenza) and those contacted were informed about the research objectives and the importance of their participation. There was no time limit for deciding whether to participate. After acceptance, telephone interviews were scheduled according to the participants' availability. In-depth interviews were guided by a semi-structured script, validated by three PhD professors, with open questions that addressed the following areas: persistence of symptoms after COVID-19 disease; access to health services; barriers and facilitators to monitoring health services; and challenges for the future. Additional questions were also used to explore and clarify themes, such as “Tell me more about this”; “Can you give me an example?”

The interview script was modified during data collection according to the emergence of new themes, meeting the framework's assumptions [36].

The interviews were carried out by a team of seven researchers, nurses, and postgraduate students, who received specific training for the activity (which totaled 40 h). Thus, interviewer effects on participants were avoided. Likewise, none of the researchers were involved in the actual care of the subjects. Field notes were taken during and after the interviews. The interviews lasted between 20 min and 1 h, with an average duration of 40 min, and were audio recorded and transcribed in full.

To guarantee that quotes were translated correctly, they were first translated into English and then back into Portuguese. Sample extracts were numbered according to the order of the interview (1–66), the length of symptomology (long or persistent COVID), and the type of service used (private or public).

### 2.4 Data analysis

Data collection and analysis were carried out simultaneously, which enabled the constant development of concepts about the data and allowed the deepening of new concepts [37]. Coding followed three stages: first, data was coded inductively, line by line, highlighting important concepts and phrases; then, focused coding highlighted the connections between categories; and finally, selective coding highlighted the central category and its relationships with other categories. The constant comparison between the coded concepts generated the central category, identified through an iterative process over several meetings with co-investigators. The theory emerged based on the central category, which comprised the main phenomena. The generation of the theory was carried out through discussions with the other researchers involved in the study, using memos and diagrams [37].

The MaxQDA® software was used to support, assist, and explore qualitative data during the initial and focused coding stages.

### 2.5 Study rigor

The research met the methodological rigor of the adopted framework by Charmaz [36] using the following criteria: credibility, originality, resonance, and usefulness. Regarding credibility, the researchers were trained to carry out the interview. Interviews were carefully transcribed, with detailed notes about the researcher's perceptions during data collection. Furthermore, the constant comparative method was used to validate the information and guarantee the study's credibility. Triangulation was reached via team discussions of the data. The use of memos and research in the scientific literature contributed to the researcher's reflexivity process, producing original discoveries. Moreover, we used creative and flexible data analysis techniques to identify novel patterns and themes. To avoid response bias, we framed questions in a third-person perspective, resulting in more truthful answers, and used open-ended questions to prompt participants to reflect and expound on their responses, preventing them from simply agreeing or disagreeing [44]. Resonance was achieved through data consistency, whereby each participant recognized and agreed with the interpretation presented, giving meaning to our findings. Following the constructivist perspective, the researcher's reflexivity was the basis throughout the process in the design of the study, in the collection and analysis of data, as well as during the writing of the findings. The research team included nurses with clinical experience in managing people living with chronic conditions (FRDMM; WB; AG) and experts and researchers in qualitative health research with a constructivist epistemological background (CL; MAS; LG; HFG).

Finally, usefulness criteria evaluated whether the study could improve people's daily lives and whether findings could be disseminated and applied to practical contexts and have real-world relevance.

### 2.6 Ethics

The study was conducted and assessed in adherence to the principles outlined in the Declaration of Helsinki. The research protocol received approval from the Research Ethics Committee of Maringá State University - UEM, under protocol number 4.518.26. Verbal informed permission, including agreement of audio recording, was acquired before conducting each interview. The participants were duly notified of their prerogative to discontinue their involvement in the study at any given point. Participation was voluntary and participants received no kind of compensation.

## 3 Results

### 3.1 Sample description

The sample included 66 people, aged 24–88 years old. There was a predominance of males (n = 44), white ethnicity (n = 45), living with family members (n = 52), more than 8 years of schooling (n = 39), and development of severe acute infection due to COVID-19 (n = 37). The most frequent symptoms affected the musculoskeletal, respiratory, integumentary, sensory, cognitive, and mental systems. Most participants developed three to four symptoms of Long COVID (n = 29) lasting between 12 weeks and 24 weeks (n = 52). Table 1 depicts an overview of the individuals included in the research.

**Table 1:** Sociodemographic and clinical description of participants (n = 66).

Variables	Users of public healthcare service (n = 28)	Users of private healthcare service (n = 38)
**Age (years)**		
Mean ± SD (range)	57.39 ± 13.60 (33–88)	50.26 ± 12.31 (24–86)
**Sex**		
Male	14 (50.00)	30 (78.95)
Female	14 (50.00)	8 (21.05)
**Ethnicity**		
White	13 (46.43)	32 (84.21)
Black/African descent	15 (53.57)	6 (15.79)
**Live alone?**		
No	20 (71.43)	32 (84.21)
Yes	8 (28.57)	6 (15.79)
**Education (years)**		
<8 years	9 (32.14)	18 (47.37)
≥8 years	19 (67.86)	20 (52.63)
**Acute COVID-19 severity**		
Moderate (Medical Unit)	18 (64.29)	11 (28.95)
Severe (Intensive Unit)	10 (35.71)	27 (71.05)
**Symptoms of Long COVID**		
Shortness of breath/Tiredness	10 (35.71)	18 (47.37)
Pain	10 (35.71)	11 (28.95)
Hair loss	5 (17.86)	7 (18.42)
Sadness/Anxiety	6 (21.43)	6 (15.79)
Memory loss	7 (25.00)	4 (10.52)
Loss of appetite	6 (21.43)	4 (10.52)
Smell change	5 (17.86)	4 (10.52)
Taste change	2 (7.15)	6 (15.79)
Motor change	2 (7.15)	3 (7.90)
**Number of symptoms**		
1–2	6 (21.42)	6 (15.79)
3–4	11 (39.29)	18 (47.37)
5–7	11 (39.29)	14 (36.84)
**Symptom duration**		
>12 and ≤24 weeks (Long COVID)	22 (78.57)	30 (78.95)
>24 weeks (Persistent COVID)	6 (21.43)	8 (21.05)

### 3.2 Overview of theory: managing long COVID symptoms and accessing health services

The iterative process between collecting and analyzing data resulted in a theoretical model. Through data analysis, we identified three categories: “Living with and managing persistent symptoms”; “Between enduring disruptive symptoms and deciding to seek professional help”; and “Tension in access to health services”. These categories interact with each other and converge on the core category of this study: “The limbo of Long COVID sufferers: between the persistence of symptoms and access to health services” (Table 2).

**Table 2:** Examples from the analysis process – from quotes to categories.

Finding units of meaning (examples of direct quotations)	Synthesizing and arriving at categories and subcategories	Core Category
P58 (Persistent Covid, Private user): It even gets in the way of sex. I can't breathe. I can't finish because I'm so tired. It feels like I'm going to die from my heart, it feels like my heart is beating inside my head. P5 (Persistent Covid, Public user): Everything I'm going to do; I need to write it down. Whether at home or work, I write down what I need to do that day.	(1) Experiencing and managing persistent symptoms	The limbo of Long COVID sufferers: between the persistence of symptoms and access to health services.
P24 (Long Covid, Public user): My body got better, what didn't get better was my head [silence], but there's no exam for that. P49 (Long Covid, Private user): This shortness of breath: the doctor says that there is a scar on my lung, that I just need to monitor it, not much can be done. Even if I wanted to see a doctor, it's not easy because the distance from my house to the hospital is far.	(2) Between enduring disruptive symptoms and deciding to seek professional help	
P27 (Persistent Covid, Public user): I was referred to the psychiatrist by the BHU doctor. I waited a year for a consultation. P51 (Long Covid, Private user): My God, I have been spending a lot on medicine. Because the plan covered hospitalization, but not medication. I can't pay my bills. P52 (Long Covid, Public user): I received very good care, both when I was hospitalized and after discharge. The nurses always called me to check on me and see if my symptoms were improving. There are physiotherapy students who come here too, they helped me a lot, they are very nice. So, I feel like I'm well taken care of. P42 (Long Covid, Private user): I receive calls from nurses. They ask me how I am, and if I need them to schedule an appointment. When I was released from the hospital, they kept calling to see if I was better. Sometimes they spoke to me, sometimes to my son or my husband … to ask, I think, whether they needed anything, if they were okay too.	(3) Tension in access to health services i) Barriers to access ii) Facilitators to access	

The fluctuating symptoms that characterize Long COVID cause survivors to self-manage their health conditions and remain ambivalent bearing these symptoms or seeking health care. This feeling reflects a lack of confidence in the ability of healthcare services to meet their post-COVID-19 needs. For those whose healthcare proved adequate to their needs, the post-COVID-19 reorganization of their lives was satisfactory. For those whose healthcare was limited, the path to survival remained unresolved. In this sense, Long COVID sufferers remain in limbo between the persistence of symptoms and access to a healthcare system capable of serving their biopsychosocial dimensions.

#### 3.2.1 Experiencing and managing persistent symptoms

Participants reported the unpleasantness of the disease's persistent symptoms. Many reported having an active life before COVID. However, with the persistence of physical symptoms (e.g., pain, difficulty breathing, fatigue), they described a significant reduction in their ability to satisfy basic and instrumental human needs.

P57 (Persistent Covid, Private user): My life has changed a lot. I still have pain in my stomach and legs. So, I had to change my diet to try to find some food that wouldn't hurt me. The pain in my legs is what bothers me the most, as it hurts so much and I can't walk much.

P31 (Long Covid, Public user): Before Covid, I didn’t have that much pain. After COVID, I have pain and wake up at night crying; I've never had that.

Although physical symptoms are widely discussed, Long COVID also affects mental health, particularly a decrease in cognitive reserve and an increase in intrusive ruminative thoughts associated with loss and disability.

P35 (Long Covid, Private user): I was very forgetful, and I wasn't like that. In my work, I am responsible for finances and I am afraid of forgetting to pay the bills. Sometimes, I arrive to do something and forget what I was supposed to do.

P21 (Long Covid, Private user): My forgetfulness has gotten a lot worse. I have always been careful with my medications. I take 14 pills in the morning and 14 at night. When night arrived, I would look in the box and there were the morning pills.

To deal with cognitive changes and resume their daily tasks, participants managed symptoms through the following strategies: day-to-day planning, reminders in visible places, and family support.

P20 (Long Covid, Private user): Everything I'm going to do has to be written on the fridge, so I don't forget.

P6 (Long Covid, Public user): My wife has to check my medicines every day to see if I took them all. When it's an important thing, like going to the doctor, she also goes along because otherwise, I won't remember everything I need to say, and then I won't remember everything the doctor told me to do.

Additionally, participants reported experiences of emotional suffering such as anxiety, nervousness, sadness, and panic. These symptoms emerged as a self-regulation strategy in the daily confrontation with the impact produced by Long COVID. There is added uncertainty regarding the duration of symptoms, the fear of not being able to recover the previous functional status, and the possibility of relapse.

P12 (Long Covid, Private user): It's a lot of nervousness, a lot of anxiety, we gain more weight, eat more, and talk less. I ended up changing more things. I no longer have patience.

P13 (Long Covid, Public user): I always had a normal life before Covid. But now, even after I've been sick for so long, it's affecting me because I can't do what I used to do. I just want my life to go back to normal.

P9 (Persistent Covid, Private user): I already had panic syndrome, but it was under control. Now, I'm worse. I'm very afraid of catching Covid again, even if I'm vaccinated because I almost died. I confess that I was a little paranoid, my children said that … because I didn't want to leave the house or receive people. So, I stayed like that for a long time, alone and scared.

Self-blame for having been diagnosed with COVID-19 was an important source of emotional suffering. Feelings of guilt arose because of questions such as “What did I do to catch COVID-19?” or “Why me, if I took such good care of myself?”

P2 (Persistent Covid, Public user): I keep thinking “Where did I get this disease?” I didn't leave the house, I sanitized all my purchases, I wore a mask, I still don't understand where I got it. It was very sad when I received the diagnosis because I saw so many of my friends who never stopped leaving the house and having their social life.

P29 (Long Covid, Private user): My children were very angry with me when I got Covid because not even they came to see me so, to avoid giving me anything; but the neighbor came. And I believe that she was the one who passed it on to me, as she was the only person I welcomed into my home. So, I was really upset because I did everything right. My children even did the shopping, so I didn't have to leave the house […] and I got it anyway.

Despite having survived the acute infection, Long COVID changed several aspects of the lives of those affected. Some participants reported individual and relational losses associated with COVID, namely social death or loss of relationships that define a person's condition.

P23 (Persistent COVID, Private user): It was such a devastating disease in the lives of so many people, including mine, that I still haven't fully recovered. I feel like a little of me was lost.

P45 (Persistent Covid, Private user): It's still very complicated for me because my wife passed away from Covid. We caught (the disease) together, but I survived, and she didn't. So, for me, it's very difficult [silence] because a part of me left with her.

P14 (Long Covid, Public user): My biggest frustration is at work, that's what makes me saddest. I can no longer work the way I worked before, I can no longer produce what I used to, and that for me is death.

People presented different ways of coping with Long COVID. Participants highlighted the following strategies to manage persistent physical symptoms associated with functional dependence: family support network and a gradual increase in work activities.

P33 (Long Covid, Public user): It's been about two weeks since my son stopped coming to bathe me every day. Now he only comes once a week, because slowly I started taking showers on my own. I feel like I'm slowly getting stronger. My husband stays with me, so I hold on and take a shower sitting down so I won't fall.

P10 (Long Covid, Private user): I had to stop working at the time [acute phase of the disease]. But I started to come back little by little […] I couldn't go back to working eight hours a day like I used to, I came back with 4 hours so I could adapt again.

On the other hand, the management of persistent emotional symptoms was facilitated by specialized mental health support and the reorganization of life priorities, which generated positive feelings of coping with the disease.

P15 (Long Covid, Private user): I am being monitored by the psychiatrist and now I am 80% better than before. I understand that I probably won't have the life I had before, but it doesn't have to be bad because of that, I just need to adapt. So, the treatment has helped me with this emotional part.

P59 (Long Covid, Private user): I’ve changed a lot. I was a person who worked too much, I didn't have time for anything. And when I got sick, my whole family stopped to take care of me … I still need care to this day, just less now. Today, I worry more about my family and my health … and enjoy life a little more.

#### 3.2.2 Between enduring disruptive symptoms and deciding to seek professional help

The experience of Long COVID symptoms presented itself as an ambivalent process between bearing the symptoms and deciding to seek professional help. This ambiguity was more evident in participants who presented intermittent symptoms, as the feeling of false recovery between episodes of worsening symptoms generated emotional stress and fear. Long COVID goes beyond the physical dimension and involves a biographical disruption. Thus, some participants expressed dissatisfaction with healthcare services, given their focus on the disease's physical symptoms and neglect of the other human dimensions, in clear discontinuity with attention to a person's ecology and circumstances.

P18 (Persistent Covid, Private user): I'm fine in life. What's not fine is my psychology. But we go to the doctor, and he sees that we have improved and says that this feeling will pass.

P56 (Long Covid, Public user): I know that I survived COVID and that my symptoms have improved, but I feel a sadness deep down, I don't know what it is […] But I never told the doctor that, because he told me to keep it in check and see that everything is fine.

As Long COVID symptoms affect daily activities, some participants reported the practice of self-medication (e.g., painkillers) as a strategy to alleviate symptoms in an attempt to regain control of their lives. At the same time, the media were responsible for disseminating false or incomplete information (as occurred with off-label hydroxychloroquine), contributing to a distrust in health services.

P60 (Persistent Covid, Public user): We know that there is no medicine for Covid. We saw that even they had difficulty knowing what to give us. It was the same with Chloroquine, some (doctors) gave it, others didn't [ …]. The pain I have, he (the doctor) said it might go away with time. So, when it's really bad, I take pain medication.

P11 (Long Covid, Public user): […] because we don't need a prescription to buy pain medication, but I try not to take it every day, only when I can't get out of bed anyway.

The heterogeneity of symptoms and the undulating course of the disease generate, on the one hand, a feeling of frustration and uncertainty in patients and, on the other, the devaluation of professionals' opinions regarding the disease's evolution. During the pandemic, health professionals went through a period of discredit; the effectiveness of their professional conduct was questioned by many. This contributed to indecision among participants about seeking professional clinical help, even during Long Covid.

P36 (Long Covid, Public user): I didn't go to the doctor because we see that not even they (the professionals) really know what to do with us. There is still a lack of information about how to take care of these symptoms that have not disappeared.

P48 (Long Covid, Public user): I see that today, in addition to having a vaccine, doctors already know how to take better care of this disease, there is no longer an exorbitant number of deaths. But I still have remnants of the disease. They just say that I should keep monitoring this, that they still don't know when they will go away and if they will go away.

The impact of symptoms on daily tasks helped participants identify the need for professional help. However, the physical distance to services to monitor post-COVID health conditions may have contributed to delays in seeking professional help and diagnosing Long COVID.

P55 (Long Covid, Public user): When I was discharged from the hospital, I was only monitored by the physiotherapist and pulmonologist. But soon they discharged me […] and this pain, this depression, was not in their area for me to talk with them about, so I had no one to talk to. It was only when I really didn't even want to get out of bed that my daughter made a direct appointment with a psychiatrist. The problem was access because we lived far from the city.

#### 3.2.3 Tension in access to health services

Healthcare services were sought when the sick person desired to return to their pre-Covid health condition. Participants experienced barriers to access but also identified facilitating aspects.

##### 3.2.3.1 Barriers to access

The experience of Long COVID led people to return to services in the hope of finding answers to their current conditions. Then, two scenarios occurred: 1) patients were referred by public primary care to specialized care, or 2) patients sought private services voluntarily.

In the first scenario, the lack of professionals and the long waiting time to obtain specialized care from the public service were experienced as barriers to the adequate implementation of Long COVID care.

P41 (Long Covid, Public user): I no longer went to the Basic Health Unit [BHU] because the doctor who accompanied me had left. He attended me for two years and now he is no longer there. But we go there and it's always the same story because not even they (the doctors) know what to do with us.

P34 (Long Covid, Private user): When I was discharged from the hospital, the doctor even asked me to follow up at the BHU to see how I would evolve. But I had to go back to work quickly, I looked for private care because at the BHU we have to wait a long time to get care.

After the hospitalization period, participants reported the need to maintain multidisciplinary monitoring at home or as an outpatient. The transition of care to ensure primary services presented gaps. Many participants experienced a discontinuity in the monitoring of their health conditions by the public health system, compared to those who used private services.

P50 (Persistent Covid, Private user): I have never received any call from the BHU, nor a visit from the healthcare team since I was discharged. Maybe they prioritize those without health insurance. As I have insurance, I did everything according to plan.

P46 (Long Covid, Private user): They (BHU professionals) called me the first week I was discharged to find out how I was doing. But I never received a medical visit, so I looked for a private one.

The reductionist role of public services was evident in the narratives of some participants who indicated that they only used the basic service to renew prescriptions, mischaracterizing the role of the service in coordinating care.

*P30 (Long Covid, Public user): Now* (with Long COVID) *I continue going to the psychiatrist and the physiotherapist. It's been over a year since I've been to the BHU* (Basic Health Unit). *When I need medicine, my wife goes and asks the doctor for the prescription and brings the medicine home.*

P61 (Persistent Covid, Private user): I no longer need to go to BHU for consultations because now all my Covid monitoring is with private doctors.

When patients sought private services voluntarily, their waiting time did not appear to be a barrier. However, the financial implications arising from Long COVID and the need for monitoring were barriers to using private services, with participants accruing debt or needing family support.

P66 (Persistent Covid, Private user): I went to several specialists (pulmonologist, cardiologist, physiotherapist …) and did several tests too. So, we ended up spending more because it wasn't planned, and we had to get money from where there was none.

P22 (Long Covid, Private user): I'm in debt because I spent so much, there's nothing left to spend.

P16 (Long Covid, Private user): I'm not ashamed to say that I needed (financial) help from my family. Because even if you pay for the plan, there are some consultations and exams that you also need to pay for. And then there is the medication.

In the early phase of the pandemic, the lack of evidence about the medium and long-term effects of Long COVID was one of the reasons that generated disbelief among participants. Health professionals also lacked adequate answers to give patients about their conditions, resulting in uninformed decision-making. A new disease, with chronicity under investigation by the scientific community, generated uncertainty among participants about how much they could benefit from health care.

P62 (Persistent Covid, Private user): I won't go, I have nothing to do at the doctor's. At the beginning (of the symptoms) I went, did a lot of tests, and the doctor himself said nothing else could be done. The tests that were carried out revealed nothing. This fatigue, this tiredness, this ease of catching the flu, there is no test to see what it is.

P28 (Long Covid, Public user): I was at the doctor six months ago and I complained about this problem of tiredness. He told me that this was normal and that it would pass. However, the fatigue and shortness of breath continue. So, I don't know if there will be a cure for this.

P40 (Long Covid, Private user): We go to the doctor but they themselves say that this will pass with time, but they don't know when. But from what I see out there, on the internet too, there is a lot that still needs to be discovered about this disease.

##### 3.2.3.2 Facilitators to access

The universality of the Unified Health System (UHS) was considered a facilitating factor, even for those individuals able to use private services. The provision of a public and universal healthcare system is a State duty, to provide healthcare for all, regardless of their purchasing power.

*P3 (Long Covid, Private user): I have a private health plan, but I know I can use* UHS *for everything. However, I saw the news on TV that the* UHS *was already full of people … so I used the private plan to let the* UHS *operate.*

*P17 (Long Covid, Public user): I'm very tired, my diabetes is out of control. Thus, I found a physiotherapist and a neurologist, I had an electrocardiogram, all through the* UHS*. It has helped me with everything I need. SUS is the best health plan in the world.*

The discoveries about Long COVID and the resulting professional action were crucial for people with Long COVID to feel better supported by services. Professional performance focusing on humanization and care was cited as helping to overcome the obstacles surrounding the disease.

P62 (Persistent Covid, Private user): My symptoms are still strong, but now the doctor himself told me that he knows more about Covid. Before, I think they were lost on how to take care of us because everything was new. I see that today they know more and, God willing, I will get well again.

P63 (Long Covid, Public user): Health workers always came to my house to ask me how I was, if I was okay, if I was getting better.

Regardless of the type of health services (public or private), participants reported the importance of health and access to services in the hope of improving their current conditions.

P53 (Long Covid, Private user): I had a lot of consequences, I didn't take care of myself before. Now I go to the doctor straight away, do all the follow-ups. The doctor referred me to therapy, and it has helped me a lot.

P32 (Long Covid, Public user): I have had a lot of support from the nearby UBS. I have now become a father and mother of a teenage son (after my wife passed away from Covid). So, I've been monitoring my health and so has he, because it's been a blow for everyone. The psychologist really says that our life is not over, that we need to continue. I still have sequelae but thank God I'm alive and healthy enough to be able to take care of my son. Now it's just me and him.

## 4 Discussion

As far as we know, this is the first Brazilian qualitative study on the experiences of people with Long COVID and how they perceive the access and support offered by existing health services. First, the findings refer to the consequences of persistent symptoms of Long COVID. Second, the trajectory of the disease required Long COVID sufferers to reorganize their routines and develop adaptive strategies. Lastly, individuals suffering from Long COVID conveyed a diverse array of both positive and negative interactions inside the healthcare system. These experiences provide valuable insights that can shape novel services or modify current ones, specifically to address the needs of this significant patient population.

Recovery from Long COVID does not follow a well-defined pattern. Most people return to their regular activities in less than 3 months, but clusters of symptoms can persist for longer periods, impairing daily activities [10]. According to the available evidence, persistent symptoms of dyspnea and fatigue are associated with the most serious manifestations of COVID-19 [45]. This justifies signaling these symptoms in many of our study's participants since we only covered the disease's more severe forms.

Our findings reveal that persistent symptoms hindered the return to pre-COVID-19 normality and, therefore, impacted the ability to satisfy basic and instrumental human needs. The study carried out by Davis et al. [46] with 3762 Long COVID patients identified that around 22% did not return to work, and around 45% were forced to reduce working hours due to disease-related complications [46]. In contrast, Garrigues et al. [47] demonstrated that health recovery was satisfactory in those who had a professional activity before the infection, reinforcing the importance of resuming daily routines after COVID-19.

Experiencing persistent symptoms of COVID-19 in a fragile healthcare system challenges the ability of survivors to restore their well-being [17], and negative experiences with healthcare services generate dissatisfaction [48]. As in previous studies, our findings highlight concerns related to “the lack of knowledge, information, and understanding about Long COVID among healthcare professionals” [26] (p.6), reiterating the need for this gap to be recognized by the scientific community [32,48]. The literature must include these experiences via the perspective of inequality and structural analysis, considering the COVID-19 pandemic's impact on both health and socioeconomic conditions. This crisis, frequently referred to as a "syndemic pandemic," underscores the need to examine how access to resources and opportunities is influenced by existing inequalities and structural determinants [49].

The ambivalence felt by people with Long COVID between their expectation of receiving adequate support and their experience when contacting the healthcare system appears to have “a direct effect on their mental and emotional state, often leading to uncertainty about what to do about their symptoms” [26] (p.5). According to Surapaneni et al. [10], rehabilitation and therapeutic treatment are necessary to reverse psychological symptoms and ensure a return to normality. In parallel, more integrated healthcare models are necessary to support patients with Long COVID and improve their health outcomes. Our findings signaled that the weaknesses of health systems are related to descriptions of how caring for Long COVID symptoms could benefit people. In this sense, resilient health systems are necessary to ensure efficient surveillance and effective responses to future health emergencies [50]. The fact that patients desire personalized and multidisciplinary services is congruent with the heterogeneous and multidimensional nature of Long COVID [51,52]. Existing models for other chronic illnesses indicate that peer support for Long COVID should extend beyond biological objectives and use the potential of relationship support and collective advocacy [19].

Our findings highlight that those participants who had satisfactory care and access to health services felt listened to and supported by health professionals. They also verbalized a need for multidisciplinary teams that can treat them holistically [37,48]. Developing countries, such as Brazil, deal daily with poverty, other neglected tropical diseases, and restricted access to primary health care [8]. Therefore, post-COVID-19 multidisciplinary care and creating health centers specialized in rehabilitation is still a challenge [8,53].

Professional expertise and knowledge have shaped the understanding of the disease, despite a lack of training and well-defined protocols for managing people with persistent symptoms. Based on pre-existing knowledge experienced in everyday life and the exchange of interprofessional experiences–so-called social constructionism–, the provision of assistance has met the needs of most people [54]. In our study, participants who benefited from satisfactory care reported the importance of the centrality of care.

To resolve Long COVID symptoms without the support of a healthcare team, people resort to self-medication. During the COVID-19 pandemic, the pattern of medication consumption in Brazil drew attention to the availability of the “covid-kit”: a combination of medications unsupported by conclusive scientific evidence [55]. A systematic review on self-medication in COVID-19 reports that the most used medications in low- and middle-income countries, such as Brazil, included “antibiotics, chloroquine or hydroxychloroquine, paracetamol, vitamins or supplements, ivermectin and ibuprofen” [56] (p.1). Self-medication to control the persistent symptoms of Long COVID indicates these medications are easy to acquire without a medical prescription. This also highlights the gap in monitoring the health of Long COVID sufferers who, because they feel abandoned by services, resort to self-medication, as verified by our findings.

In the present study, economic barriers were also potential contributors to health inequality in Long COVID, something previously identified in the literature [57]. Long waiting times and lack of access to primary care challenged participants' ability to navigate healthcare services and contributed to decreased trust in the system and healthcare professionals [17].

From a socio-ecological perspective, COVID-19 affected individual, community, and political levels, justifying the need for differentiated responses [58]. The cascade of persistent Long COVID symptoms caused biographical disruption and motivated the need for adaptation by participants. The onset of the disease affected daily life and created personal coherence, forcing individuals to mobilize new resources to deal with chaos, and thus adjust to the disease's chronicity [17]. Long COVID's persistent symptoms also highlighted the fragility of health services in providing adequate rehabilitation of these people's physical symptoms, but also in providing social, economic, and family support, domains that were also affected by COVID-19.

### 4.1 Study limitations

Given its epistemological nature, this research does not purport to establish generalizations applicable to all individuals experiencing Long COVID. In addition, our data's transferability is limited to a single area of Brazil, as it may not reflect the reality in different Brazilian states. Nevertheless, the limited number of participants, the rigorous methodology used, and the iterative nature of the study all contributed to the trustworthiness of the findings.

Furthermore, given the prevailing state of alert, interviews by telephone were deemed the most suitable approach to ensure the safety of all participants by mitigating the need for travel. This interview method, while potentially introducing bias due to the absence of non-verbal cues and decreased spontaneity, was chosen to reliably and vividly capture the authentic experiences and emotions of the participants. The aim was to minimize the occurrence of false memories or other errors that may arise over time. In addition, while interviewers repeated questions during the interview, some participants presented difficulty remembering or reporting past events or experiences accurately, maybe due to memory distortions provoked by the long-term effects of COVID-19.

Although this study recorded several participant characteristics, it did not consider social disparities that may affect the Long COVID trajectory. Factors such as comorbidities, cultural diversity, housing conditions, income, and health inequalities could not be related to our findings but might influence personal experiences. Nevertheless, comprehensive representation in forthcoming endeavors is essential to guarantee resilient guidance. Additionally, there is a pressing need to incorporate firsthand experiences of marginalized communities affected by Long COVID.

Participants classified as having mild COVID-19 in the acute phase were omitted. Based on previous evidence, this population is less relevant for assessing the repercussions of Long COVID. However, future studies should seek to understand the effects of Long COVID in this population. Although all participants in our study had Long COVID, their symptoms varied in duration, which may affect their perceptions regarding the disease's impact on their living conditions and health.

### 4.2 Implications for practice

Primary healthcare professionals (HCPs) often serve as the first point of contact for patients and have a vital position as gatekeepers in enabling patient access to secondary care, as well as evaluating patient needs. Hence, enhancing the availability of basic healthcare services is essential to ensure that individuals with Long COVID get more assistance and appropriate referrals. Pervasive distrust demands consideration when examining the accessibility and use of healthcare services. Individuals with Long COVID expressed concerns about structural obstacles within the healthcare system, as these had a detrimental effect on their capacity to obtain and avail themselves of necessary assistance. As previously proposed, enhanced intercommunication across disparate healthcare providers is needed to facilitate improved provision of follow-up assistance by HCPs, in conjunction with augmented training and education for HCPs regarding post-COVID conditions. The accessibility and delivery of services are clearly being affected by broader structural difficulties that may be traced back to years of austerity measures. There is a prevailing concern over the limited accessibility of assistance within a system that is now experiencing a state of overwhelming demand. Although COVID-19 has exposed the structural vulnerabilities of the Brazilian Unified Health System, it also revealed the strengths of the largest public and universal health system in the world, which plays a leading role in monitoring and providing healthcare [59], as well as the reorganization of rehabilitation and articulation of actions to combat Long COVID.

However, there is a need for greater communication and knowledge about Long COVID in the political and assistance spheres [26]. The heterogeneity of symptoms and the consequences experienced by survivors indicate a need for multidisciplinary services [50] that provide a holistic, person- and family-centered assessment, with appropriate treatments and referrals to specialized services when necessary. Limited access to primary health care and the lack of rehabilitation programs after discharge can represent additional challenges that make it difficult to manage persistent symptoms in the long term [8]. Furthermore, within the scope of public policies, a national plan should be developed for people with Long COVID based on organizing access to primary and specialized services for this condition.

Additional qualitative research in culturally diverse samples of people with Long COVID is recommended to address local health needs. As services for people with Long COVID are developed, their views and experiences should continue to be explored.

## 5 Conclusion

This constructivist GT provides a theoretical perspective about the lived limbo of Long COVID sufferers as a tension between the persistence of symptoms and access to health services. For some, managing symptoms and re-adapting to their daily activities was well supported by health services. For others, the lack of professional support resulted in a fragile resumption of pre-COVID-19 activities, with repercussions on social, family, domestic, work, and economic life. Our findings indicate a need to strengthen primary and specialized health services, public and private, with a well-defined line of care for accessing and monitoring the health of Long COVID sufferers.

## Ethical statement

The study was conducted in accordance with the Declaration of Helsinki, and approved by the State University of Maringá Ethics Committee in Research with Human Beings (Opinion 670 No. 4.518.26). Informed consent was obtained from all subjects involved in the study. Participation in the study was completely voluntary and anonymous. Participants received no compensation.

## Data availability statement

Not applicable.

## Funding

This work is supported by national funds through FCT—Fundação para a Ciência e a Tecnologia, I.P. (UIDB/05704/2020 and UIDP/05704/2020) and under the Scientific Employment Stimulus-Institutional Call (https://doi.org/10.54499/CEECINST/00051/2018/CP1566/CT0012, accessed on December 30, 2023).

## CRediT authorship contribution statement

**Francielle Renata Danielli Martins Marques:** Writing – review & editing, Writing – original draft, Visualization, Validation, Software, Resources, Methodology, Investigation, Formal analysis, Data curation, Conceptualization. **Carlos Laranjeira:** Writing – review & editing, Writing – original draft, Visualization, Validation, Supervision, Project administration, Methodology, Investigation, Funding acquisition, Formal analysis, Conceptualization. **Lígia Carreira:** Writing – review & editing, Methodology, Investigation. **Adriana Martins Gallo:** Writing – review & editing, Methodology, Investigation. **Wanessa Cristina Baccon:** Writing – review & editing, Methodology, Investigation. **Herbert de Freitas Goes:** Writing – review & editing, Visualization, Investigation. **Maria Aparecida Salci:** Writing – review & editing, Writing – original draft, Visualization, Validation, Supervision, Project administration, Methodology, Investigation, Funding acquisition, Data curation, Conceptualization.

## Declaration of competing interest

The authors declare the following financial interests/personal relationships which may be considered as potential competing interests:

Carlos Laranjeira reports financial support was provided by FCT—Fundação para a Ciência e a Tecnologia, I.P.. Carlos Laranjeira reports a relationship with FCT—Fundação para a Ciência e a Tecnologia, I.P. that includes: funding grants. If there are other authors, they declare that they have no known competing financial interests or personal relationships that could have appeared to influence the work reported in this paper.
